# Supplementary material for: Chromothripsis during telomere crisis is independent of NHEJ, and consistent with a replicative origin
Source: Genome Res. 2019 May;29(5):737–49. doi: 10.1101/gr.240705.118 (PMC6499312; doi:10.1101/gr.240705.118)
Supplement: Supplemental Material [file supp_gr.240705.118_Supplemental_file_1.zip › contigs/annotated_contigs/DB57/contig.2.DB57_length_681_mean_cov_12.6196769457.docx]

**DB57_length_681_mean_cov_12.6196769457**

```
GAGAAAGGAAAGTAAATGTGGCCAGGAGGAGCTGAGTATTCAGCCCTCAAAACATTGCTTACTATACCCTATCTACTGTGCATATGTGT
 >chr17:46249607-46250017 - E=6e-232
GTATATATGTGTGAGTGAGAGAGTGGGAAAAGAGCATGGTGGTAGTGAAAAGAATAACGAGTGTATTTTTCTTCACTTTGCCCCATGTA

TAAAATAGCTAAATATGATTCTGGTACACATATTTACATTATAAGGGCCCAATTCCTATTTTATCGGGTAATGTGACGAGATGAAACCG

GTTAATGGGAGTAAGAAACTTAGGCCAATTATGTTATGACTTTGACCGTATAAACATTTGACTGGAGTTAGACACTGGCATTGAGTTAG

AACCCATTGTGAATCTACGGCAATTAATTAAAAGACCATTCCAGCTACCC|TCAG|CTTCTCTTAATTAAGAAACAATCAGGCCCAGTG
                                                   >chr17:46291687-46291962 - E=1e-147
CAATGGCCTATGCCTGTAATTCCAGCACTTGGCAGGCCAAGGTGGGTAGATTGCTTGAACCCAAGAGTTTGACACCAGCCTGGGCAACA

TGGCAAAATCCCATCTCTACAAAAAATTAAAGAAGAAGAAACAATCAGAAGCTAATAGGAAGCTGTAATTAAAGTTTGATATGTTTTAG

AGGAGATGAAGAAGAAGGCAATACATCCTTTGGGCTGGTCAGTGGTATTTTCTAGTGAAT
```
